# Individual Differences in Political Ideology and Disgust Sensitivity Affect Real-Time Spoken Language Comprehension

**DOI:** 10.3389/fpsyg.2021.699071

**Published:** 2021-10-11

**Authors:** Isabell Hubert Lyall, Juhani Järvikivi

**Affiliations:** Department of Linguistics, University of Alberta, Edmonton, AB, Canada

**Keywords:** psycholinguistics, spoken language comprehension, semantic anomalies, gender stereotypes, political ideology, disgust sensitivity, speaker identity, pupillometry

## Abstract

Individuals' moral views have been shown to affect their event-related potentials (ERP) response to spoken statements, and people's political ideology has been shown to guide their sentence completion behavior. Using pupillometry, we asked whether political ideology and disgust sensitivity affect online spoken language comprehension. 60 native speakers of English listened to spoken utterances while their pupil size was tracked. Some of those utterances contained grammatical errors, semantic anomalies, or socio-cultural violations, statements incongruent with existing gender stereotypes and perceived speaker identity, such as “I sometimes buy my bras at Hudson's Bay,” spoken by a male speaker. An individual's disgust sensitivity is associated with the Behavioral Immune System, and may be correlated with socio-political attitudes, for example regarding out-group stigmatization. We found that more disgust-sensitive individuals showed greater pupil dilation with semantic anomalies and socio-cultural violations. However, political views differently affected the processing of the two types of violations: whereas more conservative listeners showed a greater pupil response to socio-cultural violations, more progressive listeners engaged more with semantic anomalies, but this effect appeared much later in the pupil record.

## 1. Introduction

Language comprehension is a complex process: The listener has to identify the sounds in a word, recognize the word form, retrieve information associated with this form (Cutler and Clifton, 1999; Sahin et al., 2009), and integrate the word into the rapidly unfolding context within a few hundreds of milliseconds (Hagoort and Indefrey, 2014; Levinson, 2016) in an incremental fashion (Altmann and Kamide, 1999; Sedivy et al., 1999; Kamide et al., 2003; Hagoort and Van Berkum, 2007). At the same time, a body of research has shown the importance of context, broadly understood, to real-time language comprehension. For example, an utterance at odds with factual knowledge about the state of affairs in the real world, such as “Dutch trains are *white*” when they are in fact yellow, triggers an N400 signature at the target word *white* (Hagoort et al., 2004; Hagoort and Van Berkum, 2007) in the same way as a semantically anomalous word (“Dutch trains are *sour*”). Further, a statement about a peanut being salted can trigger this same N400 response if embedded in a (fictional) story context that does not warrant an inanimate interpretation (Nieuwland and Van Berkum, 2006). Important, both speaker-related attributes, for example unfamiliar accents (Porretta et al., 2016; Grey and van Hell, 2017; Porretta and Tucker, 2019; Arnhold et al., 2020) or inferred gender (Van Berkum et al., 2008), as well as listener-based individual differences (Van den Brink et al., 2012; Hubert Lyall and Järvikivi, 2021) have been found to affect language comprehension ease. In this paper, we investigate whether two further individual difference factors, an individual's propensity to disgust as well as their political views, are associated with their language processing performance.

Disgust is an emotional response to contagious and unsafe situations, such as pathogens or bodily secretions, that is intended to protect an organism from pathogen contamination (Neuberg et al., 2011; Schaller and Neuberg, 2012). Importantly, the feeling of disgust extends beyond simple pathogen avoidance to include *moral* disgust or purity violations (Wagemans et al., 2018), for example triggered by bad moral character (Giner-Sorolla and Chapman, 2017; Molho et al., 2017), socially deviant behavior associated with out-groups, or the out-groups themselves (Schaller and Neuberg, 2012; Murray and Schaller, 2016; Dawydiak et al., 2020). Out-group members effectively end up being treated like infected in-group members (Petersen, 2017). Further, disgust is closely associated with the *Behavioral Immune System*, whose goal it is to direct attention to a threat of pathogen contamination, in an effort to trigger behaviors that will prevent those pathogens from entering the body (Neuberg et al., 2011; Murray and Schaller, 2016; Aarøe et al., 2017).

Commonly, disgust sensitivity is higher for women than for men, and for individuals scoring higher in the Big Five traits of Agreeableness, Conscientiousness, and Neuroticism, and lower in Openness (Druschel and Sherman, 1999). Since disgust is easily triggered not just by the presence of actual pathogens, but also by members of a perceived out-group or concepts considered immoral, thresholds for disgust have been shown to predict socio-political attitudes: People who have strong negative attitudes toward socially deviant groups tend to be disgusted more easily, and are more likely to identify as conservative (Faulkner et al., 2004; Inbar et al., 2009; Smith et al., 2011; Schaller and Neuberg, 2012; Hodson and Dhont, 2015; Murray and Schaller, 2016; Tybur et al., 2016; Aarøe et al., 2017). This link between disgust sensitivity and an individual's political ideology has been traced back to notions of purity and conformity, which, in ancestral populations, may have served the purpose of keeping novel pathogens, that the in-group would have no antibodies against, at bay (Haidt and Graham, 2007; Inbar et al., 2009). Disgust thus shapes an individual's world view, attitudes, and behavior, even down to voting behavior in political elections (Park, 2015; Shook et al., 2017, 2019; Karg et al., 2019; Stewart et al., 2020).

Thus far, there is no systematic analysis of the influence of disgust sensitivity on real-time language comprehension. However, there is some evidence that individual's moral and political views affect their language processing behavior. Van Berkum et al. (2009) showed that linguistic processing cost is not just modulated by factual states of the world, but also by a person's beliefs. They measured participants' ERP responses to statements such as “I think euthanasia is an acceptable vs. unacceptable course of action.” Crucially, their participants either identified as voters of a conservative party or not. They found that words inconsistent with the participants' moral values elicited an early ERP positivity between 200 and 250 ms and a subsequent N400 after the onset of the clashing word (“acceptable” vs. “unacceptable,” depending on the individual's party denomination).

Participants' political ideology has been also shown to affect how they understand inferred causal relations in events involving interpersonal verbs such as *admire, envy*, or *punish*. Marrville (2017) showed that participants' political views significantly predicted whether participants completed sentences fragments such as “Melissa discouraged Sean because.” by continuing with the first or the second NP (Melissa or Shawn). More precisely, Marrville (2017) showed that the completions interacted with affective properties of the verbs, namely their valence and dominance, and the participants' political ideology. For example, more conservative participants tended to have more NP1 continuations for low dominant verbs when they were also high valence (thank), but more NP2 continuations when the verb was low valence (criticize). However, this pattern was reversed for more liberal participants. Recently, Niemi et al. (2020) replicated the overall pattern for political views in a similar set-up, suggesting that individuals' moral binding values such as of loyalty, obedience to authority and purity correlated with explicit causal judgments of the NP2 in the event as more likely to have allowed—and perhaps deserved—any harmful outcomes of a particular action. Together these studies suggest that individuals' political ideology is connected to how they interpret interpersonal events and, further, that it might be associated with stronger victim-blaming tendencies in more conservative-leaning participants (see also Niemi and Young, 2016).

Relatedly, recent research suggests that language comprehension is affected by listeners' personality traits that have been linked to moral and political views and disgust sensitivity. Van den Brink et al. (2012) showed that more empathetic listeners showed a larger N400 response to (socially) unusual statements (such as a male adult saying “I cannot sleep without my *teddy bear*”) than their less empathetic counterparts. Hubert Lyall and Järvikivi (2021) showed in a pupillometry study that listeners' Big-5 personality traits systematically modulated listeners' real-time language processing in similar scenarios. For example, less open participants showed greater pupil dilation when processing spoken utterances with morpho-syntactic errors (“He frequently have burgers for dinner”), and more introverted listeners showed greater pupil dilation in response to both semantic anomalies (“I often read heads for pleasure” vs. books) and socio-cultural clashes (“I sometimes buy my bras at Hudson's Bay,” vs. “ties,” spoken by a male speaker). More generally, these results suggest that listener-internal factors, such as moral and political views and personality traits, interact with information inferred about the speaker and affect ERP and pupillary responses to stimuli during real-time language processing (see also Van Berkum et al., 2008; Hanulíková et al., 2012; Porretta et al., 2016; Grey and van Hell, 2017).

In the present study, we will investigate the extent to which individuals' disgust sensitivity predicts their language processing behavior when listening to statements that include various degrees of deviance from linguistic and social norms and stereotypes. Furthermore, we will compare the effects of individuals' disgust sensitivity and political ideology. Answers to disgust sensitivity scale might reflect the respondent's moral and political views more indirectly than answers to political ideology questionnaires which can be subject to conscious manipulation due to the somewhat obvious nature of the questions.

We used pupillometry to investigate the processing of sentences containing socio-cultural clashes, semantic anomalies, and morpho-syntactic errors. Pupil size is considered an indicator of autonomous nervous system activity in humans (Steinhauer et al., 2004; Gingras et al., 2015). Specifically, it has been shown to reflect resource allocation (Rondeel et al., 2015) due to cognitive effort, workload, arousal, attention, or affect (Hess and Polt, 1960, 1964; Ahern, 1978; Ahern and Beatty, 1979; Just and Carpenter, 1993; Goldinger and Papesh, 2012; Gingras et al., 2015; Winn et al., 2018). For linguistic stimuli, pupil size has been shown to be responsive to orthographic errors (Thierfelder et al., 2020), intelligibility (Zekveld et al., 2010), ambiguity (Vogelzang et al., 2016; Winn et al., 2018), grammatical gender mismatches and semantic anomalies (Demberg and Sayeed, 2016), accentedness (Porretta and Tucker, 2019), complexity (Ahern, 1978; Ben-Nun, 1986; Just and Carpenter, 1993; Engelhardt et al., 2010), and individual difference factors (Ahern, 1978; Ahern and Beatty, 1979; Lõo et al., 2016; Hubert and Järvikivi, 2019). Importantly, the pupillometry paradigm does not require participants to complete an overt task or action, reducing the possibility of task effects.

We tracked the participants' pupil size as they listened to anomalous or unusual statements, and correlated changes in pupil size with participants' conservatism and disgust sensitivity scores. We were especially interested in pupillary changes in response to socio-cultural violations, statements that are at odds with common gender stereotypes. Stereotypes are cognitive shortcuts (Hilton and von Hippel, 1996) that are considered part of world knowledge (Carreiras et al., 1996) and activated immediately during language comprehension (Banaji and Hardin, 1996; Osterhout et al., 1997; Pyykkönen et al., 2010; Hanulíková and Carreiras, 2015; Molinaro et al., 2016). Importantly, the extent to which individuals engage in stereotyping depends on aspects of their identity (Sibley and Duckitt, 2008; Akrami et al., 2011; Quadflieg and Macrae, 2011).

In our study, socio-cultural violations rely on *vocal gender*, that is, the speaker's gender as inferred from their voice. Voices with lower formant frequencies, a lower fundamental frequency, and greater resonance are typically interpreted as male (Strand, 1999; Ko et al., 2006). Vocal gender has recently been found to affect the comprehension of segments containing stereotypically male or female occupations (Grant et al., 2020). We expected more disgust-sensitive individuals to allocate more resources, as indicated by a larger pupil size, to anomalous stimuli; specifically, we expected higher disgust sensitivity and conservatism scores to be correlated with a stronger response to socio-cultural clashes, due to a stronger tendency to stigmatize.

## 2. Methods

### 2.1. Participants

82 participants completed the main experiment and post-tests for this study, which received research ethics committee approval from Research Ethics Board 2 at the University of Alberta. While non-native speakers of English were allowed to participate in the study, their data (*n* = 14) was not used in the analyses reported below. Data from participants whose comprehension question accuracy was below 80% (*n* = 8) were removed, as attention to and comprehension of the experiment stimuli could not be guaranteed in those cases (min = 75%; max = 100%; mean = 93:7%, 95% CI [92.1, 95.3]). Trials during which more than 33% of data points were recorded as missing data, i.e., N/A, were excluded from analyses (n = 782; 8% of trials). The analyses in this paper are thus based on the data obtained from 60 native speakers of English (*male/female* = 8/52; *age min/max* = 17/83, mean = 24.9, 95% CI [21.5, 28.3]). Of those 60 participants, 38 were recruited from the University of Alberta undergraduate linguistics student pool and received course credit for their participation; the remaining 22 participants were recruited from the general population, with no restrictions on age or background, and received a small monetary compensation for their participation.

A two-tailed *t*-test showed that the age distribution differed significantly between the two recruitment strategies [mean_external_ = 33.5, 95% CI [25.3, 41.7]; mean_internal_ = 19.9, 95% CI [19, 20.8]; *t*_(21.48)_ = −3.21, *p* = 0.004], with externally recruited participants being significantly older and their age distribution being much wider.

### 2.2. Materials and Design

We created 240 utterances for experimental stimuli (see Table 1; the full list of stimuli in Supplementary Materials). While the focus of our paper is on the processing of socio-cultural violations, morpho-syntactic errors and semantic anomalies were included to compare these pragmatic deviations, which violate common expectations given the context, to more language-internal, structural and semantic-level violations. Both morpho-syntactic errors and semantic anomalies have been shown to result in processing delays for reading and listening times (Just and Carpenter, 1980; Braze et al., 2002; De Vincenzi et al., 2003; Ditman et al., 2007; Tokowicz and Warren, 2010), as exemplified in augmented P600 and N400 ERP amplitudes, respectively (Kutas and Hillyard, 1980; Ni et al., 1998; Braze et al., 2002; Hagoort and Indefrey, 2014), but also in larger pupil dilation (Beatty, 1982; Engelhardt et al., 2010; Demberg and Sayeed, 2016; Zekveld et al., 2018).

**Table 1:** Overview of stimuli and experimental conditions.

	**Clash type**	**# of stimuli**	**Clash description**	**Example stimulus**
Controls	Socio-cultural	120	Clash with the speaker's perceived Identity as per common gender stereotypes	*I usually buy my **bras** at Hudson's Bay*, spoken by a male speaker.
	Morpho-syntactic	56	Violation of subject-verb agreement	*She usually **drive** her car slowly in the snow*.
	Semantic	32	Semantic mismatch between the verb and the object	*People often read **heads** for pleasure at night*.
	Filler	32	N/A - non-anomalous	*Chickens normally live in a coop*.

All utterances followed the same syntactic pattern to ensure comparability across regions (Jegerski and VanPatten, 2013). For item recording, items were presented to one male and one female native speaker of Western Canadian English in random order, and recorded in a sound-treated booth using a *Korg MR-2000S* studio recorder with a *Countryman E6* earset microphone. Item recordings in which the prosody sounded noticeably different from those of other items were re-recorded with the speaker. Utterances were then distributed across four lists, counterbalanced for error condition (non-anomalous baseline vs. anomalous) and speaker gender (male vs. female). Each list included the same 32 filler utterances, resulting in 135 utterances per list. Each participant was presented with one list (and, accordingly, each item only once, in just one condition and spoken by one speaker).

Additionally, all items were previously rated for acceptability in a separate, off-line Likert-style ratings experiment, by a separate set of participants (see Hubert Lyall, 2019; 99 native speakers of English recruited from the pool of undergraduate linguistics students at the [University of Alberta]; male/female = 59/40 (60%/40%); age min/max = 17/31; mean = 20.4 years). The resulting average per-item ratings were fed into the statistical models reported below as a numerical predictor.

In the main experiment, a comprehension question was presented to the participant after approximately 30% of items (i.e., each participant was presented with a question after 38 to 41 items total). Questions were 128 simple *yes/no* questions in line with well-established world knowledge, such as “Do giraffes have long necks?” after the unrelated filler item “Giraffes always have very long necks,” to check for both attention to the experiment, and comprehension of the auditory stimuli that were presented (De Vincenzi et al., 2003; Hanulíková et al., 2012).

### 2.3. Procedure

After introducing the participants to the experimental setup, they were seated in an adjustable chair in a dimly lit experiment booth at the Centre for Comparative Psycholinguistics, University of Alberta. Light levels were kept constant throughout the experiment and for all participants. Participants were asked to place their head on a chinrest for additional stability, and to ensure a constant screen-to-eye distance. They were then instructed to follow the instructions on the screen to calibrate the eye-tracker, and to complete the experiment. During the experiment, the pupil size of the participant's right eye (cf. Kahneman and Beatty, 1966; Porretta and Tucker, 2019) was recorded at 250 Hz using an *EyeLink 1000* system on a desktop PC.

Each trial began with a one-point drift correct, immediately followed by the display of a fixation cross at the center of the screen. Pupil size was recorded from the start of the fixation cross. Two thousand milliseconds later, the audio stimulus began to play, and pupil size was recorded until 500 ms after audio offset. After an inter-stimulus interval of 3,000 ms, to allow pupil dilation to return to baseline, the next trial began. Participants were given longer breaks approximately every thirty-five trials; the length of these longer breaks was up to the participant. The main experiment took between 20 and 30 min to complete. Participants then moved on to the post-tests described below.

### 2.4. Post-questionnaires

Participants completed three post-test questionnaires after the main experiment session, so as not to prime them toward the purpose of the study. To assess the influence of disgust sensitivity on language comprehension, the *Disgust Scale - Revised* [*DS-R*; (Haidt et al., 1994) modified by Olatunji et al. (2007)], which was also used in, for example, Inbar et al., 2009, 2012; Ahn et al., 2014; Hubert and Järvikivi, 2019, was administered to participants. Participant's political views were assessed using a Wilson-Patterson-type test (Wilson and Patterson, 1968), the full version of which can be found in section 1 (Supplementary Material). This test was also chosen for results to be directly comparable to recent research involving political values and disgust sensitivity (Jost et al., 2003; Smith et al., 2011; Ahn et al., 2014; Hatemi and Verhulst, 2015). Note that the Wilson-Patterson scale is a conservatism scale; as such, high scores signify a conservative outlook. Both the political questionnaire and the DS-R were coded in *E-Prime* 2.0 (Psychology Software Tools Inc., 2012). In addition, data on the participants' language background, including proficiency in other languages and places the participant had lived, was collected via a pen-and-paper language background questionnaire.

Some studies have found a link between higher disgust sensitivity and a conservative world view (Graham et al., 2009; Inbar et al., 2009, 2012; Murray and Schaller, 2016; Aarøe et al., 2017); within our participant sample, we observed a weak, non-significant (*p* > 0.05) trend in the same direction, that is, for individuals with higher disgust sensitivity to also be more conservative (*r* = 0.22, *p* = 0.1) (Supplementary Figure 1). We did not observe significant differences in the distributions of either political views or disgust sensitivity scores between men and women; however, externally recruited participants were significantly less disgust sensitive than their internally recruited peers [mean_external_ = 1.71, 95% CI [1.52, 1.9]; mean_internal_ = 2.16, 95% CI [1.97, 2.35]; *t*_(53.92)_ = 3.25, *p* = 0.002].

## 3. Results

### 3.1. Data Pre-processing and Model-Fitting

The raw pupillometry data was pre-processed in R (Version 3.6.3, R Core Team, 2020) and RStudio (Version 1.3.959, RStudio Team, 2020), with one data point being one pupil size sample. Blinks and the adjacent 20 data points (10 to the left, 10 to the right) were removed, and the onset of each target word was centered at 0 ms. Baseline pupil sizes were calculated per participant per trial over the time period of −200 ms until onset of the target word, and data points further than 2.5 SD's from the respective participant-trial baseline (3% of total data points) were removed.

All results reported below were obtained through generalized additive mixed effects modeling (*GAM modeling*, or *GAMM*) using the mgcv (Version 1.8-28, Wood, 2011) and itsadug (Version 2.3, van Rij et al., 2017) packages in R, with relative pupil size as the dependent variable. GAM modeling is well suited to time-series data, such as pupillometry, as it is able to capture non-linear interactions between continuous predictors without losing information in time-binning (Tremblay and Newman, 2015; van Rij et al., 2019). All models included a random smooth for participant by time, and a random intercept by item to account for individual differences within the stimuli, and for random variance between participants beyond the factors of interest. Average item rating, time (−200 to 2,000 ms from target word onset), and disgust sensitivity and political ideology scores were tested as fixed predictors. Visualizations were produced using the itsadug, ggplot2 (Version 3.2.1, Wickham, 2016), and ggpubr (Version 0.3.0, Kassambara, 2020) packages. Data in the time window from 200 ms before target word onset to 2,000 ms after was analyzed. The models were fitted using a forwards step-wise selection procedure, where the inclusion of variables was evaluated using a combination of a χ^2^ test of REML scores via the compareML() function, visual inspection, and the estimated p-value of the smooth parameter via the report_stats() function (see e.g., Porretta and Tucker, 2019; van Rij et al., 2019).

### 3.2. Morpho-Syntactic Errors

Changes in pupil size in response to morpho-syntactic errors were modeled as a control condition (see Supplementary Table 1 for the model output). The results showed significant non-linear effects of Time, Item Rating, and and interaction between the two (all *p*'s < 0.0001). Items that contained a morpho-syntactic error were associated with significantly larger pupil dilation (Supplementary Figure 2), suggesting that the pupillometry paradigm was able to track changes in pupil size associated with a processing difficulty at an erroneous word. Individual disgust sensitivity and political views were tested as model predictors, but were not found to be associated with significant effects on pupil dilation in this condition.

### 3.3. Semantic Anomalies

**Figure 1:**
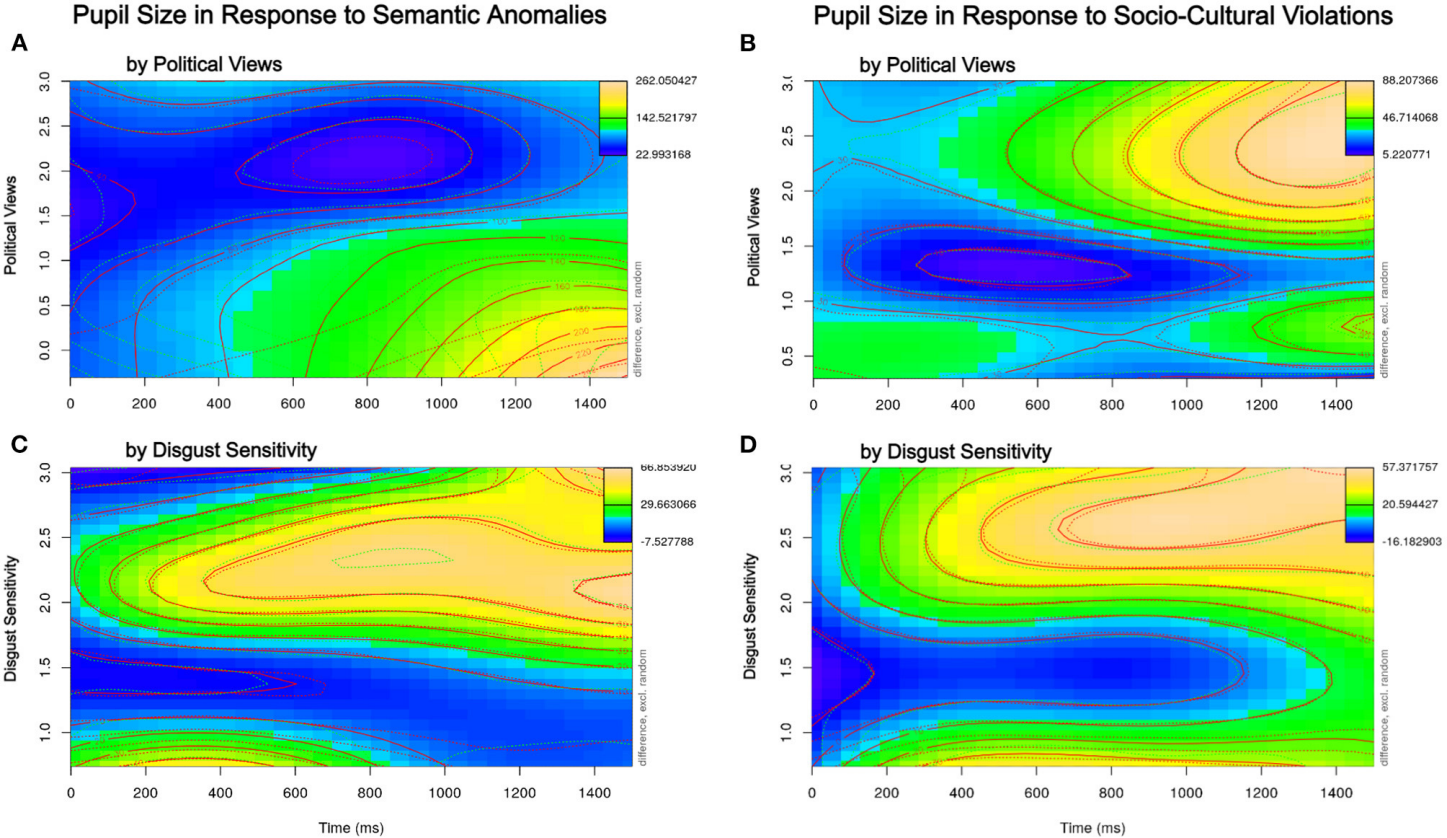
Difference in pupil size in anomalous/clashing conditions compared to non-anomalous/non-clashing conditions by Political Views and Disgust Sensitivity (y-axis) and Time from the onset of the target word (x-axis) for semantic anomalies (**A,C**, respectively) and socio-cultural clashes (**B,D**, respectively).

The model output is depicted in Supplementary Table 2. The results showed significant non-linear effects of Time, Item Rating, and an interaction between these (*p* < 0.0001). In addition, we found a significant interaction between Disgust Sensitivity and Time (*p*'s < 0.0001) and, importantly, a three-way non-linear interaction between Disgust Sensitivity, Time, and Item Rating (*p*'s < 0.0001). Additionally, there was a significant interaction between Political Ideology and Item rating (*p* < 0.0001) that was qualified by a three-way interaction with Time (*p* < 0.0001). In order to assess these interactions, in what follows, we will inspect the respective difference plots that depict the difference in pupil size between the anomalous/clashing and the non-anomalous/non-clashing conditions by Time from the target word onset (x-axis, 0–1,500 ms) and participants' Political Ideology (Figure 1A) and Disgust Sensitivity (Figure 1B) scores.

Figure 1 visualizes the three-way interaction between time since onset of the target word (on the x-axis), the respective individual difference variable (disgust sensitivity or political views on the y-axis), and clash condition (implied; see details below). The participant's pupil size is represented via a color scale: Thus, the difference in clashing vs. non-clashing conditions is implied in this type of plot; the color scale indicates the difference in pupil size when a listener encounters a clashing statement, as compared to when they encounter a non-anomalous statement. A blue color indicates a small (or even negative) change in pupil size when listening to a clashing statement (as compared to a non-anomalous statement), whereas a yellow or orange color indicates a larger dilation. We thus see that for the three-way interaction between time, the semantic anomaly condition, and political views in Figure 1A, it is more liberal listeners (lower half of the plot, y-axis) who experience a larger change in pupil dilation: there is a gradual change from blue to green color starting around 500 ms that starts turning into yellow and orange around 1,000 ms after onset of the target word.

As to the interaction with disgust sensitivity, visualized in Figure 1C, listeners with higher than average disgust sensitivity (upper half of y-axis) showed a larger pupil dilation starting already around 300ms after the onset of the target word.

### 3.4. Socio-Cultural Violations

Socio-cultural violations, as the main focus of analysis in this paper, are defined as statements violating common expectations regarding what a speaker of the perceived gender would be expected to say, such as an adult male saying “I cannot sleep without my teddy bear in my arms.” The final model output can be found in Supplementary Table 3. The results are depicted in Figures 1B,D. Firstly, we found significant effects of Time and Item Rating, the latter suggesting that statements clashing with the speaker's perceived gender identity indeed elicited larger pupil dilation than the non-clashing ones (*p*'s < 0.0001). Secondly, there were significant interactions between Item Rating and both the listener's political views and their disgust sensitivity (*p*'s < 0.0001) that further interacted with time (*p*'s < 0.0001). As Figure 1 shows, in both cases, it was listeners with high scores on the respective questionnaires, that is, more conservative-leaning and more disgust-sensitive listeners, respectively, who experienced a larger increase in pupil dilation in response to a socio-cultural violation.

## 4. Discussion

Prior research suggests that individual's moral views (Van Berkum et al., 2009) as well as aspects of personality (Van den Brink et al., 2012; Hubert Lyall, 2019; Hubert Lyall and Järvikivi, 2021) predict online spoken language comprehension times. In this research, we asked whether and to what extent person's sensitivity to disgust and their political ideology affect the processing of spoken utterances with different types of violations/clashes.

We found a significant effect of item rating—the extent to which a statement was grammatically, semantically, or pragmatically anomalous—for all three violation types. In all cases, listeners showed a significant pupil dilation in the clashing condition as compared to baseline, reflecting an increased processing cost as indicated by increased pupil dilation (Hess and Polt, 1960, 1964; Ahern, 1978; Ahern and Beatty, 1979; Just and Carpenter, 1993; Goldinger and Papesh, 2012; Gingras et al., 2015; Winn et al., 2018). Importantly, listeners' conservatism and disgust sensitivity scores interacted with item rating: More disgust-sensitive and conservative individuals allocated more resources to the processing of socio-cultural violations, suggesting that an individual's disgust sensitivity modulated their allocation of cognitive resources for understanding the pragmatic implications of the message. This result extends the findings from, for example, Schaller and Neuberg (2012); Murray and Schaller (2016); Aarøe et al. (2017); Wagemans et al. (2018) to the realm of real-time language comprehension, suggesting that the behavioral immune system influences an individual's response to a statement as soon as the statement is being processed.

While prior research has found that more disgust-sensitive individuals generally tend to be more conservative (Faulkner et al., 2004; Graham et al., 2009; Inbar et al., 2009, 2012; Smith et al., 2011; Schaller and Neuberg, 2012; Hodson and Dhont, 2015; Murray and Schaller, 2016; Tybur et al., 2016; Aarøe et al., 2017), the two variables were not strongly correlated in our participant sample; instead, we observed only a weak, non-significant correlation. This suggests that, even though the two variables may occasionally be correlated, their influence on real-time language comprehension may be distinct.

Further, with the proviso that pupillometry is not the best method to track precise processing time course, our results do suggest that the effect of the listener's political views had a later onset than that of disgust sensitivity. Thus, disgust sensitivity may affect processing earlier, perhaps affecting anticipation or immediate integration into the context.

We observed an interesting result in the processing of semantic anomalies. While more disgust-sensitive listeners experienced increased pupil dilation shortly after a semantically anomalous word, it was *less* conservative listeners that experienced greater resource allocation to a semantically anomalous stimulus.

The late onset of the effect in progressive listeners is in line with and could be analogous to the late posterior positivity/P600 reported in Kuperberg et al. (2020), reflecting an initial failure to integrate the anomalous utterance into the existing situation model. In this scenario, even though progressive listeners are initially not affected by the anomalous expression, they experience increased processing load at a later, integration, stage. The late posterior positivity/P600 component is also often found in response to unexpected syntactic structures, animacy violations, or semantically anomalous words, sometimes reflecting late, coherence establishing processes (Osterhout and Holcomb, 1992; Kuperberg et al., 2006, 2020). While the component generally peaks between 500 and 800 ms after stimulus onset, some prior research (see, for example, Kim and Osterhout, 2005; Nieuwland and Van Berkum, 2005) has found later peaking, around 900 to 1,000 ms after stimulus onset. Given the make-up of our stimuli, this effect would coincide time-wise with the end of the experimental utterance and so with late integration.

Finally, while we found a pupil response to all three types of deviations from the norm, only the processing of semantic anomalies and socio-cultural clashes was affected by participants' political ideology and disgust sensitivity. This suggests that there may have been an emotional component to our results. Pupillometry has been shown to be sensitive to effects stemming from both increased processing load as well as from affective demands (Beatty, 1982; Steinhauer et al., 2004; Gingras et al., 2015). Research has shown that language processing is affected by mood, and happy and sad mood might engage different neural networks (Egidi and Gerrig, 2009; Egidi and Nusbaum, 2012; Egidi and Caramazza, 2014). Moreover, statements conflicting with an individual's values have been shown to quickly engage the affect system and modulate language comprehension (Van Berkum et al., 2008, 2009). Relatedly, Van Berkum et al. (2013) showed that individuals' mood affects the extent to which they engage in predictive processing during language comprehension. However, this does not seem to be the case across-the-board: they found no effect of mood for agreement violations, only for the violations of expectations as prompted by utterances depicting interpersonal events with social causes and consequences. Since both political views and disgust sensitivity have been linked to personality traits (Haidt et al., 1994; Druschel and Sherman, 1999) that themselves affect the processing of spoken statements (Hubert Lyall and Järvikivi, 2021), it may be that, at least partly, these effects stem from the same underlying emotional response (van Berkum, 2020; Wheeler et al., 2020).

In summary, our study is the first to show that an individual's disgust sensitivity, the emotional signature of the Behavioral Immune System, along with the individual's political views, modulate the resources allocated to the comprehension of an auditory linguistic message. Individuals' politically leaning toward conservatism showed more resource allocation to socio-cultural violations, which aligns with prior literature suggesting that this group is more prone to elevated Behavioral Immune System activity, and out-group stigmatization (see Hodson and Dhont, 2015, for an overview). Specifically, our results are the first to show that the effects of disgust sensitivity on the comprehension of a deviating statement, via the immediate inference of vocal gender and linguistic meaning, are immediate and automatic. Our results further highlight that individual difference variables do not affect language comprehension wholesale, across the board; but that these variables interact with the type of clash being processed. The results thus support theories of language comprehension that assign importance to extra-linguistic context, consisting not only of linguistics context proper but also of individual's knowledge and conceptions of the world, and in which this extra-linguistic context influences real-time language comprehension early on. Our results further suggest that both an individual's experience with the world as well as (related) aspects of their personality may shape their real-time language processing.

## Data Availability Statement

The raw data supporting the conclusions of this article will be made available by the authors, without undue reservation.

## Ethics Statement

The studies involving human participants were reviewed and approved by Research Ethics Board 2 at the University of Alberta. The patients/participants provided their written informed consent to participate in this study.

## Author Contributions

IH and JJ jointly conceived and designed the experiments and contributed to the writing of the manuscript. IH analyzed the data, wrote the first full draft, and performed the experiments with the assistance of undergraduate assistants. All authors contributed to the article and approved the submitted version.

## Funding

This research was supported by a Social Sciences and Humanities Research Council of Canada (http://www.sshrc-crsh.gc.ca/) Partnership Grant (Words in the World, 895-2016-1008).

## Conflict of Interest

The authors declare that the research was conducted in the absence of any commercial or financial relationships that could be construed as a potential conflict of interest.

## Publisher's Note

All claims expressed in this article are solely those of the authors and do not necessarily represent those of their affiliated organizations, or those of the publisher, the editors and the reviewers. Any product that may be evaluated in this article, or claim that may be made by its manufacturer, is not guaranteed or endorsed by the publisher.
